# Roles of metabolic dysregulation in osteoarthritis

**DOI:** 10.3389/fcell.2026.1840208

**Published:** 2026-05-29

**Authors:** Deliang Cheng, Rui Li, Chao Liu, Changming Zheng, Xiaolong Du

**Affiliations:** Department of Hand Surgery, Honghui Hospital, Xi’an Jiaotong University, Xi’an, China

**Keywords:** AMPK, glucose metabolism, lipid metabolism, metabolic dysregulation, mTOR, osteoarthritis

## Abstract

Osteoarthritis (OA) is increasingly recognized not merely as a mechanically driven degenerative disorder, but as a complex whole-joint disease shaped by profound metabolic dysregulation. Beyond cartilage wear, OA involves coordinated pathological changes in subchondral bone, synovium, and periarticular tissues, with metabolic syndrome emerging as a major systemic driver of disease initiation and progression. This review summarizes current advances in the understanding of metabolic disturbances in OA, with a particular focus on lipid, glucose, bone, and calcium–phosphate metabolism. In lipid metabolism, adipokines such as leptin, cholesterol accumulation, peroxisome proliferator-activated receptor signaling, and altered polyunsaturated fatty acid balance contribute to chronic low-grade inflammation, chondrocyte catabolism, and cartilage degradation. In glucose metabolism, hyperglycemia, insulin resistance, and advanced glycation end products aggravate oxidative stress, inflammatory signaling, and extracellular matrix destruction, thereby accelerating structural joint damage. Bone metabolic remodeling, including dysregulation of the OPG/RANKL/RANK axis, osteocalcin signaling, hyaluronic acid turnover, CTX-II release, and matrix metalloproteinase activation, further disrupts the balance between bone formation and resorption and amplifies cartilage degeneration. In parallel, calcium–phosphate metabolism influences OA progression through calcitonin, parathyroid hormone, and vitamin D-dependent regulation of osteochondral homeostasis. Collectively, these findings indicate that OA is driven by dynamic crosstalk among systemic metabolism, inflammatory mediators, chondrocyte dysfunction, and subchondral bone remodeling. A deeper understanding of these interconnected metabolic pathways not only refines the pathogenic framework of OA but also identifies potential therapeutic opportunities, including metabolic modulation, biomarker-guided stratification, and multi-target intervention strategies aimed at slowing disease progression and improving patient outcomes.

## 1 Introduction

Osteoarthritis (OA) is an inflammatory joint disease primarily characterized by the degeneration or loss of articular cartilage and involving multiple joint structures, including articular cartilage, subchondral bone, ligaments, the joint capsule, and synovium (Abramoff and Caldera, 2020). According to etiology, OA can be classified into primary and secondary forms. Primary OA occurs in the absence of an obvious initiating factor, and its pathogenesis remains incompletely understood, with aging and obesity representing the major risk factors. Secondary OA, by contrast, may be induced by trauma, mechanical stress, congenital abnormalities, metabolic disorders, and other causes. Despite these etiological differences, both forms share common metabolic mechanisms during disease progression, in which mechanical joint loading, inflammatory mediators, and immune responses cooperatively drive cartilage destruction. These processes are, in turn, influenced to varying degrees by systemic metabolic factors, thereby further aggravating bone metabolic disturbances and accelerating cartilage degeneration.

Metabolic syndrome (MS) comprises a cluster of disorders, including obesity, dyslipidemia, hypertension, and impaired glucose metabolism, and may predispose individuals to type 2 diabetes, cardiovascular disease, and fatty liver disease (Mendrick et al., 2018). The association between MS and OA may be attributable to its interactions with basal inflammation, adipokines, and mechanical loading. Obesity promotes OA through increased biomechanical stress on the joints as well as adipokine-mediated chronic low-grade systemic inflammation. Dyslipidemia contributes to cartilage damage through lipid accumulation, leading to reductions in bone density and bone mass. Type 2 diabetes has likewise been associated with the development of OA, underscoring the important contribution of hyperglycemia to disease pathogenesis.

Under OA conditions, cartilage and related joint tissues undergo metabolic dysregulation as a consequence of abnormal mechanical stress, elevated inflammatory mediators, and excessive innate immune activation, thereby further accelerating disease progression. In chondrocyte metabolism, decreased activity of adenosine 5′-monophosphate-activated protein kinase (AMPK) and silent mating type information regulation 2 homolog 1 (SIRT1), together with increased activity of mammalian target of rapamycin (mTOR), induce mitochondrial dysfunction, oxidative stress, and inflammation, ultimately impairing chondrocyte renewal, promoting apoptosis, and disrupting tissue structure and function (Berenbaum et al., 2017). Inflamed synovial tissue also produces increased levels of proteolytic enzymes and releases multiple bone resorption-related metabolites. Bone resorption and bone formation are the two major physiological processes governing bone metabolism, and their dynamic equilibrium is essential for maintaining skeletal homeostasis. Disruption of either process can disturb bone metabolic balance and thereby promote the onset and progression of OA.

## 2 Lipid metabolism in OA

Lipid metabolism contributes to the initiation and progression of OA primarily through two major mechanisms: adipokine-driven chronic low-grade systemic inflammation and cholesterol accumulation-induced cellular injury. Leptin is a protein produced by adipose tissue, and both leptin and its receptor are primarily involved in the regulation of food intake and energy expenditure, while also participating in metabolism, inflammation, and immune responses. Leptin contributes to inflammatory responses by promoting the upregulation of interleukin-1 (IL-1) and IL-6. In addition, by inducing the expression of matrix metalloproteinase 1 (MMP1) and MMP13, leptin activates multiple signaling pathways, including signal transducer and activator of transcription (STAT), mitogen-activated protein kinase (MAPK), protein kinase B, and nuclear factor-κB (NF-κB), thereby promoting the catabolic degradation of cartilage (Hui et al., 2012). Within the immune system, leptin can enhance T-cell differentiation, regulate immune function, and thereby participate in OA progression. In murine models, mice deficient in leptin or its receptor were shown not to exhibit an increased risk of OA despite severe obesity (Griffin et al., 2009), indicating that the pro-inflammatory effects of leptin outweigh the contribution of mechanical overload alone. Clinical studies have further shown that serum leptin levels are associated with OA (Kroon et al., 2019), while synovial fluid leptin concentrations correlate with radiographic severity in patients with OA (Ku et al., 2009), suggesting that leptin may serve as a useful biomarker for evaluating disease progression.

Cholesterol is another major risk factor in dysregulated lipid metabolism that contributes to the development and progression of OA. Hypercholesterolemia can trigger chronic inflammatory responses and induce mitochondrial dysfunction, leading to oxidative stress in chondrocytes and thereby promoting cartilage injury. *In vitro* studies using cultured human OA chondrocytes have shown that statins antagonize the effects of cholesterol supplementation, reduce MMP13 expression, and attenuate cartilage degradation (Simopoulou et al., 2010). In animal models of OA, statins have likewise been shown to delay disease progression (Farnaghi et al., 2017; Yudoh and Karasawa, 2010; Akasaki et al., 2009). Furthermore, a study by Clockaerts et al. (Hui et al., 2012) demonstrated that statin use is associated with reduced progression of knee OA (KOA). These findings provide a rationale for further clinical investigation of statins as a potential therapeutic strategy for OA. Peroxisome proliferator-activated receptors (PPARs) comprise three major isoforms, PPARα, PPARγ, and PPARβ/δ, which are differentially distributed across tissues and play central roles in the regulation of lipid metabolism. Among these, PPARγ is particularly involved in cholesterol-dominant lipid metabolic pathways. Through interaction with the liver X receptor (LXR), PPARγ increases the expression of ATP-binding cassette transporter A1 (ABCA1), thereby promoting cholesterol efflux from chondrocytes, preventing lipid accumulation, and protecting cartilage (Xu et al., 2011). In addition, PPARγ suppresses inflammatory responses *in vivo* and mitigates the deleterious effects of hyperglycemic conditions on chondrocytes. In the study by Zhu et al. (Zhu et al., 2019), articular cartilage from patients with OA and from mice with medial meniscal injury exhibited marked suppression of PPARγ, whereas restoration of PPARγ expression by demethylating treatment alleviated cartilage damage. Furthermore, PPARγ agonists such as oridonin and astragaloside have been shown to reduce IL-1β-mediated inflammatory responses in chondrocytes, suggesting potential therapeutic value in OA (Jia et al., 2019; Qu et al., 2017). In addition, ω-3 polyunsaturated fatty acids (PUFAs) are closely associated with cartilage composition. Dietary intake of ω-3 PUFAs may exert protective effects against OA by correcting the elevated ω-6/ω-3 ratio observed in osteoarthritic joints (Kimmerling et al., 2020; Masuko et al., 2009). However, their clinical efficacy still requires further investigation (Table 1).

**TABLE 1:** Major metabolic alterations driving osteoarthritis progression.

Metabolic alteration	Mediators	Effects	Therapeutic opportunities	Value
Lipid metabolic dysregulation	Leptin, PPARγ, LXR, ABCA1, cholesterol, ω-3 PUFAs	Promotes synovial inflammation, oxidative stress, chondrocyte catabolism, and matrix degradation	Leptin blockade, PPARγ activation, cholesterol efflux enhancement, ω-3 PUFA supplementation	Links obesity-associated metabolic disturbance to OA progression; useful for biomarker and metabolic intervention studies
Glucose metabolic imbalance	Hyperglycemia, insulin resistance, AGEs, RAGE, MAPK, NF-κB	Enhances inflammatory signaling, suppresses chondrocyte proliferation, and accelerates cartilage degeneration	Glycemic control, insulin-sensitizing agents, AGEs/RAGE-targeted strategies	Provides a mechanistic bridge between diabetes and OA; promising for phenotype-based management
Excessive bone/Cartilage resorption metabolism	CTX-II, MMP1, MMP2, MMP9, MMP13	Drives extracellular matrix destruction, cartilage erosion, and structural progression	MMP inhibition, anti-catabolic therapies, matrix-preserving strategies	CTX-II and MMP13 have value as candidate biomarkers and therapeutic targets
Folate-related metabolic disorder	Folate deficiency, homocysteine, ROS, intracellular Ca^2+^	Promotes oxidative stress, synoviocyte injury, and metabolic imbalance in joint tissues	Folate repletion and oxidative stress reduction	Represents a modifiable metabolic risk factor with potential preventive value
Adenosine metabolism and autophagy impairment	Extracellular adenosine, autophagy-related pathways	Reduces cartilage homeostasis and accelerates chondrocyte degeneration	Adenosine replenishment, autophagy-promoting interventions	Supports the development of disease-modifying strategies targeting metabolic homeostasis
Energy-sensing pathway dysfunction	SIRT1, AMPK, mTOR, NAMPT, HIF-2α	Induces mitochondrial dysfunction, inflammatory activation, apoptosis, and impaired matrix maintenance	AMPK/SIRT1 activation, mTOR suppression, metabolism-modulating agents such as metformin or resveratrol	Offers a strong mechanistic framework for precision metabolic therapy in OA

## 3 Glucose metabolism in OA

Disordered glucose metabolism contributes to the development and progression of OA. Hyperglycemia primarily promotes joint damage through oxidative stress, insulin resistance, and the accumulation of advanced glycation end products (AGEs), which collectively upregulate inflammatory mediators and hydrolytic enzymes, thereby exacerbating tissue injury (Laiguillon et al., 2015; Rosa et al., 2011). Meta-analytic evidence has demonstrated a positive association between type 2 diabetes mellitus and OA (Williams et al., 2016). Insulin resistance promotes local inflammatory responses and induces a state of chronic low-grade systemic inflammation, thereby facilitating OA progression. At the same time, insulin resistance is often considered in the context of obesity-related metabolic abnormalities, and earlier views suggested that the relationship between type 2 diabetes and OA remained controversial. Hyperinsulinemia may downregulate thyroid hormone levels, thereby inhibiting chondrocyte differentiation (Askari et al., 2017). However, the study by Al-Jarallah et al. (AL-Jarallah et al., 2016) found that the progression of osteophyte formation in patients with type 2 diabetes was attenuated following insulin therapy. In addition, a study based on a rat model of type 1 diabetes showed that combined treatment with insulin and vanadium reduced the development of diabetes-induced OA (El Karib et al., 2016).

**FIGURE 1:**
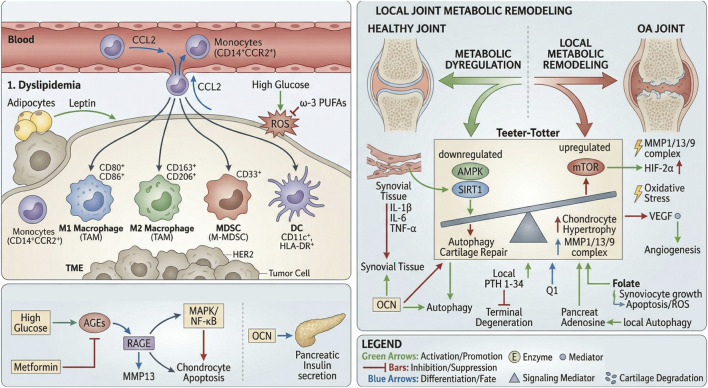
Roles of metabolic dysregulation in osteoarthritis.

AGEs are the end products of non-enzymatic glycation reactions and accumulate progressively with aging, being associated with multiple age-related degenerative disorders. In OA, AGEs accelerate cartilage degeneration by suppressing chondrocyte proliferation, increasing the levels of pro-inflammatory factors, and downregulating enzymes involved in collagen turnover and matrix degradation (Huang et al., 2009). Studies targeting the receptor for advanced glycation end products (RAGE) have shown that RAGE expression is increased in osteoarthritic cartilage, which in turn enhances MAPK and NF-κB signaling in chondrocytes and promotes the expression of MMP13 (Loeser et al., 2005). In a rabbit OA model, Li et al. (Li et al., 2016a) demonstrated that increased AGEs aggravated exercise-induced cartilage degradation, whereas pioglitazone treatment exerted beneficial effects. Moreover, AGEs inhibitors have shown efficacy in randomized double-blind studies by reducing pain and inflammation and improving joint function in patients with OA (Garg et al., 2013) (Figure 1).

## 4 Bone metabolism in OA

Bone resorption and bone formation are the two major physiological processes regulating skeletal metabolism. Under normal conditions, their dynamic equilibrium is essential for maintaining bone homeostasis. In the pathophysiological setting of OA, however, bone resorption and bone formation are differentially activated or suppressed at distinct stages of disease, thereby disrupting bone metabolic balance and accelerating OA progression.

### 4.1 Bone formation metabolism in OA

Osteoprotegerin (OPG) regulates bone resorption by competitively binding to receptor activator of nuclear factor-κB ligand (RANKL) and preventing its interaction with receptor activator of nuclear factor-κB (RANK). In early OA, RANKL predominates, leading to enhanced osteoclast activity and consequent bone and cartilage destruction. In late-stage OA, by contrast, OPG expression increases, osteoclast-mediated resorption is suppressed, and bone overgrowth becomes more prominent. Tat et al. (2011) demonstrated that strontium ranelate can reduce the resorptive activity of osteoblast-related bone turnover in human OA and suppress subchondral bone heterogeneity during OA progression. Zhang et al. (2011) further reported that tetraphosphate exerts bone-protective effects by inhibiting osteoclast activity and improving bone quality. These findings suggest that reducing bone resorption in the early stages of OA may represent a rational therapeutic strategy. Osteocalcin (OCN) is not only essential for calcium metabolism and skeletal maintenance, but also participates in anti-aging processes. OCN exerts anti-inflammatory effects by reducing the levels of pro-inflammatory mediators such as tumor necrosis factor-α (TNF-α) and IL-1. In addition, OCN may lower blood glucose by promoting pancreatic β-cell proliferation and insulin secretion, thereby reducing the accumulation of advanced glycation end products (AGEs). Adipokines such as leptin and adiponectin may also be upregulated by OCN, thus contributing to OA progression. Several studies have shown that OCN levels increase with OA progression (Kumm et al., 2013), and in patients with hand OA, OCN concentrations are positively correlated with disease severity (Kalichman and Kobyliansky, 2010). However, other studies have reported no significant association or even opposite findings (Kenanidis et al., 2011; Sowers et al., 1999), indicating that further investigation is still required.

Hyaluronic acid (HA) can promote cartilage formation and repair. Nevertheless, evidence suggests that the interaction between HA and CD44 may also promote extracellular matrix degradation and cartilage degeneration (Bourguignon, 2008). Although the precise mechanisms of HA in OA progression remain incompletely understood, studies of intra-articular HA injection have suggested that it may serve as a therapeutic option, particularly in early OA (Ricci et al., 2017; Jevsevar et al., 2015). Although both the American Academy of Orthopaedic Surgeons and the OA Research Society International do not recommend HA as a standard treatment for OA, the lack of systematic quantification and uniform criteria in many existing studies indicates that its clinical efficacy still warrants broader and more rigorous evaluation.

### 4.2 Bone resorption metabolism in OA

The C-terminal cross-linked peptide of type I collagen (CTX-I) is a degradation product of type I collagen and serves as one indicator of bone resorption. In the studies by Cibere et al. (2009); Xin et al. (2017), elevated urinary CTX-II was associated with an increased risk of KOA, and its concentration was positively correlated with OA progression. Moreover, the ratio of CTX-II to cartilage synthesis markers was shown to provide better discrimination of disease progression (Cibere et al., 2009). However, the specificity of urinary CTX-II remains limited, and it may therefore be more useful as an adjunctive biomarker combined with other markers, such as cartilage glycoprotein-40, for the early diagnosis and evaluation of OA (Wang et al., 2019). At the translational level, Park et al. (2015) developed a microcone conductive chip for measuring CTX-II in serum and urine, which may facilitate the practical clinical application of CTX-II in OA diagnosis.

MMPs are a family of proteolytic enzymes capable of degrading extracellular matrix components, and multiple MMP subtypes contribute to the breakdown of type II collagen in cartilage during OA. In Asian populations, increased expression of MMP1, MMP2, and MMP9 has been associated with OA susceptibility (Zeng et al., 2015). Upregulation of MMP13 expression further increases the susceptibility to KOA and aggravates disease severity (Sun et al., 2019). In the study by Baragi et al. (2009), selective MMP13 inhibitors showed chondroprotective effects and may also alleviate pain in a rat model of OA. In addition, histone deacetylase inhibitors have been reported to attenuate cartilage degradation in OA by downregulating MMP13 expression (Higashiyama et al., 2010).

### 4.3 Calcium and phosphate metabolism in OA

Calcium and phosphate metabolism participates in the regulation of bone formation and bone resorption by modulating the distribution of calcium and phosphate in the skeleton and circulation, thereby maintaining dynamic skeletal homeostasis. Calcitonin (CT) suppresses osteoclastic bone resorption, enhances osteoblastic activity, and promotes calcium salt deposition. In rat models of OA, CT has been shown to attenuate disease progression and reduce pain by increasing the expression of transforming growth factor-β1 (TGF-β1) (Wen et al., 2016). CT can also protect rat cartilage by blocking the action of IL-1β (Bai et al., 2019). However, two randomized, double-blind, multicenter controlled trials evaluating the efficacy of oral salmon calcitonin (sCT) in patients with OA found that its therapeutic benefit did not reach statistical significance (Karsdal et al., 2015). Nevertheless, other studies have suggested that sCT combined with ω-3 fatty acids exerts favorable effects in animal models of OA (Adeyemi and Olayaki, 2019), indicating its potential as part of combination therapy. Parathyroid hormone (PTH) is a polypeptide hormone secreted by the parathyroid glands that increases serum calcium and decreases serum phosphate levels. Treatment with PTH 1–34 may reduce terminal inflammatory chondrocyte degeneration by enhancing autophagy and may therefore exert beneficial effects in early OA (Orth et al., 2014; Chen et al., 1985; Morita et al., 2018). However, its precise mechanisms of action at different stages of OA remain to be clarified.

Vitamin D promotes the absorption of calcium and phosphate in the body and is therefore beneficial for bone formation. Vitamin D deficiency has been associated with an increased risk of KOA, whereas 1,25-dihydroxyvitamin D3 may facilitate cartilage repair by reducing the expression of MMPs. Multiple studies have supported a beneficial therapeutic role of vitamin D in OA (Manoy et al., 2017). For example, the study by Manoy et al. (2017) showed that patients with KOA experienced reduced pain and improved grip strength and physical function after treatment with vitamin D2. In addition, an observational cohort study demonstrated that after vitamin D supplementation, patients with lower serum levels of 1,25-dihydroxyvitamin D3 showed improvements in OA severity, pain scores, and overall health status (Thomas et al., 2019).

### 4.4 Other metabolic pathways in OA

Folate appears to play a contributory role in the progression of OA. Mechanistic studies have shown that folate deficiency leads to the accumulation of homocysteine, and together these factors promote oxidative stress and intracellular calcium overload. Homocysteine can additionally increase membrane-associated osteoclastic activity, reduce osteoblastic function, and directly damage the bone matrix (Herrmann et al., 2005). Hsu et al. (2016) further demonstrated that folate deficiency impairs synoviocyte growth and promotes apoptosis, whereas correction of folate deficiency reduces excessive reactive oxygen species production, decreases intracellular calcium release, and attenuates synoviocyte apoptosis. These findings support a promotive role of folate deficiency in OA progression. Adenosine-related metabolism also influences the development of OA through modulation of autophagy. In a rabbit OA model, autophagy was found to be enhanced during the early stage of disease but diminished at later stages, whereas the autophagy-specific inhibitor 3-methyladenine markedly accelerated degeneration of chondrocytes and cartilage (Cheng et al., 2017). Likewise, adenosine has been reported to delay OA progression by modulating extracellular adenosine metabolism (Haskó et al., 2018). Corciulo et al. (2017) showed that intra-articular injection of a liposomal adenosine suspension, designed to replenish adenosine, prevented OA progression in rats, whereas reduced extracellular adenosine levels accelerated disease development. These findings suggest that targeting adenosine metabolism may represent a promising strategy for the prevention and treatment of OA.

The expression of sirtuins (SIRTs) is closely associated with aging, and these proteins participate in OA progression primarily through regulation of autophagy, in part via autophagy-related gene 7. Deficiency of SIRT1 leads to chondrocyte hypertrophy and loss of extracellular matrix. Mice lacking SIRT1 exhibit more rapid OA progression (Matsuzaki et al., 2014), and clinical evidence has shown that SIRT1 expression is negatively correlated with the severity of KOA (Li et al., 2016b), supporting its protective role. Under inflammatory stress, SIRT1 in chondrocytes is cleaved into inactive N-terminal (NT) and C-terminal (CT) fragments, and the increased serum NT/CT SIRT1 ratio observed in patients with early OA suggests that this measure may serve as a potential early diagnostic biomarker (Batshon et al., 2020). Guo et al. (Guo et al., 2017) reported that melatonin, both *in vitro* and *in vivo*, suppresses IL-1β-induced MMP production by modulating SIRT1-dependent nicotinamide phosphoribosyltransferase activity and promoting deacetylation. Resveratrol has also been shown to prevent OA-related cartilage destruction by activating SIRT1 and thereby suppressing hypoxia-inducible factor-2α (HIF-2α) expression (Li et al., 2015). In addition, metformin delays OA progression in mouse models by activating the AMPKα/SIRT1 pathway and inhibiting extracellular matrix degradation (Wang et al., 2020). Collectively, these findings highlight the potential of SIRT1 as a therapeutic target in OA.

## 5 Conclusion

Accumulating evidence indicates that metabolic dysregulation is a central determinant of OA pathogenesis rather than a secondary consequence of joint degeneration. Lipid abnormalities, hyperglycemia, insulin resistance, advanced glycation end product accumulation, disrupted bone turnover, and altered calcium–phosphate homeostasis collectively shape a pathogenic network that links systemic metabolic imbalance to local inflammation, chondrocyte dysfunction, extracellular matrix degradation, and subchondral bone remodeling. These mechanisms interact dynamically across disease stages, helping to explain the marked heterogeneity of OA progression and clinical presentation. Importantly, this emerging framework broadens the therapeutic perspective of OA. Targets such as leptin signaling, PPARγ, AGEs/RAGE, OPG/RANKL, MMP13, calcitonin, parathyroid hormone, and vitamin D pathways may offer opportunities for mechanism-based intervention, while biomarkers including leptin, osteocalcin, and CTX-II may improve disease monitoring and patient stratification. However, many current findings remain preclinical or clinically inconsistent, underscoring the need for better standardized cohorts, longitudinal studies, and integrative mechanistic analyses. Overall, OA should be viewed as a metabolically influenced inflammatory joint disease involving coordinated dysfunction of cartilage, bone, and synovium. Future research integrating metabolism, immunology, biomechanics, and translational therapeutics will be essential for developing more precise and effective strategies to prevent structural progression and preserve joint function.
